# Palmprint and Face Multi-Modal Biometric Recognition Based on SDA-GSVD and Its Kernelization

**DOI:** 10.3390/s120505551

**Published:** 2012-04-30

**Authors:** Xiao-Yuan Jing, Sheng Li, Wen-Qian Li, Yong-Fang Yao, Chao Lan, Jia-Sen Lu, Jing-Yu Yang

**Affiliations:** 1 State Key Laboratory of Software Engineering, Wuhan University, Wuhan 430072, China; 2 College of Automation, Nanjing University of Posts and Telecommunications, Nanjing 210046, China; E-Mails: lisheng1989@gmail.com (S.L.); 705353627@qq.com (W.-Q.L.); yaoyf@njupt.edu.cn (Y.-F.Y.); lanchao18@gmail.com (C.L.); echosenm@gmail.com (J.-S.L.); 3 State Key Laboratory for Novel Software Technology, Nanjing University, Nanjing 210046, China; 4 College of Computer Science, Nanjing University of Science and Technology, Nanjing 210094, China; E-Mail: yangjy@mail.njust.edu.cn

**Keywords:** multimodal biometric feature extraction, palmprint and face, subclass discriminant analysis (SDA), generalized singular value decomposition (GSVD), kernel subclass discriminant analysis (KSDA)

## Abstract

When extracting discriminative features from multimodal data, current methods rarely concern themselves with the data distribution. In this paper, we present an assumption that is consistent with the viewpoint of discrimination, that is, a person's overall biometric data should be regarded as one class in the input space, and his different biometric data can form different Gaussians distributions, *i.e.*, different subclasses. Hence, we propose a novel multimodal feature extraction and recognition approach based on subclass discriminant analysis (SDA). Specifically, one person's different bio-data are treated as different subclasses of one class, and a transformed space is calculated, where the difference among subclasses belonging to different persons is maximized, and the difference within each subclass is minimized. Then, the obtained multimodal features are used for classification. Two solutions are presented to overcome the singularity problem encountered in calculation, which are using PCA preprocessing, and employing the generalized singular value decomposition (GSVD) technique, respectively. Further, we provide nonlinear extensions of SDA based multimodal feature extraction, that is, the feature fusion based on KPCA-SDA and KSDA-GSVD. In KPCA-SDA, we first apply Kernel PCA on each single modal before performing SDA. While in KSDA-GSVD, we directly perform Kernel SDA to fuse multimodal data by applying GSVD to avoid the singular problem. For simplicity two typical types of biometric data are considered in this paper, *i.e.*, palmprint data and face data. Compared with several representative multimodal biometrics recognition methods, experimental results show that our approaches outperform related multimodal recognition methods and KSDA-GSVD achieves the best recognition performance.

## 1. Introduction

Multimodal biometric recognition techniques use multi-source features together in order to obtain integrated information to obtain more essential data about the same object. This is an active research direction in the biometric community, for it could overcome many problems that bother traditional single-modal biometric system, such as the instability in one's feature extraction, noisy sensor data, restricted degree of freedom, and unacceptable error rates. Information fusion is usually conducted on three levels, *i.e.*, pixel level [1,2], feature level [3–5] and decision level [6–9]. The former two levels mainly aim at learning descriptive features, while the last level aims at finding a more effective way to use learned features for decision making. Especially, at the pixel level and feature level, discriminant analysis technique always plays an important role to acquire more descriptive or more discriminative features.

Linear discriminant analysis (LDA) is a popular and widely used supervised discriminant analysis method [10]. LDA calculates the discriminant vectors by maximizing the between-class scatter and minimizing the within-class scatter simultaneously. It is effective in extracting discriminative features and reducing dimensionality. Many methods have been developed to improve the performance of LDA, such as enhanced Fisher linear discriminant model (EFM) [11], improved LDA [12], uncorrelated optimal discriminant vectors (UODV) [13], discriminant common vectors (DCV) [14], incremental LDA [15], semi-supervised discriminant analysis (SSDA) [16], local Fisher discriminant analysis [17], Fisher discrimination dictionary learning [18], and discriminant subclass-center manifold preserving projection [19].

In recent years, many kernel discriminant methods have been presented to extract nonlinear discriminative features and enhance the classification performance of linear discrimination techniques, such as kernel discriminant analysis (KDA) [20,21], kernel direct discriminant analysis (KDDA) [22], improved kernel Fisher discriminant analysis [23], complete kernel Fisher discriminant (CKFD) [24], kernel discriminant common vectors (KDCV) [25], kernel subclass discriminant analysis (KSDA) [26], kernel local Fisher discriminant analysis (KLFDA) [27], kernel uncorrelated adjacent-class discriminant analysis (KUADA) [28], and mapped virtual samples (MVS) based kernel discriminant framework [29].

In this paper, we have developed a novel multimodal feature extraction and recognition approach based on linear and nonlinear discriminant analysis technique. We adopt the feature fusion strategy, as features play a critical role in multimodal biometric recognition. More specifically, we try to answer the question of how to effectively obtain discriminative features from multimodal biometric data. Some related works have appeared in the literature. In [1,2], multimodal data vectors are firstly stacked into a higher dimensional vector to form a new sample set, from which discriminative features are extracted for classification. Yang [3] discussed the feature fusion strategy, that is, parallel strategy and serial strategy. The former uses complex vectors to fuse multimodal features, *i.e.*, one modal feature is represented as the real part, and the other modal feature is represented as the imaginary part; while the latter stacks features of two modals into one feature, which is used for classification. Sun [4] proposed a method to learn features from data of two modalities based on CCA, but it has not been utilized in biometric recognition, and is not convenient to learn features from more than two modes of data.

While current methods generally extract discriminative features from multimodal data technically, they have rarely considered the data distribution. In this paper, we present an assumption that is consistent with the viewpoint of discrimination, that is, in the same feature space, one person's different biometric identifier data can form different Gaussians, and thus his overall biometric data can be described using mixture-Gaussian models. Although LDA has been widely used in biometrics to extract discriminative features, it has the limits that it can only handle the data of one person that forms a single Gaussian distribution. However, as we pointed out above, in multimodal analysis, different biometric identifier data of one person can form mixture-Gaussians. Fortunately, subclass discriminant analysis (SDA) [30] has been proposed to remove such a limit of LDA, and therefore could be used to describe multimodal data that lie in the same input space.

Based on the analysis above, in this paper we propose a novel multimodal biometric data feature extraction scheme based on subclass discriminant analysis (SDA) [20]. For simplicity, we consider two typical types of biometric data, that is, face data and palmprint data. For one person, his face data and palmprint data are regarded as two subclasses of one class, and discriminative features are extracted by seeking an embedded space, where the difference among subclasses belonging to different persons is maximized, and the difference within each subclass is minimized. Then, since the parallel fusion strategy is not suitable to fuse features from multiple modals, we fuse the obtained features by adopting the serial fusion strategy and use them for classification.

Two solutions are presented to solve the small sample size problem encountered in calculating the optimal transform. One is to initially do PCA preprocessing, and the other is to employ the generalized singular value decomposition (GSVD) [31,32] technique. Moreover, it is still worthy to explore the non-linear discriminant capability of SDA in multimodal feature fusion, in particular, when some single-modals still show complicated and non-linearly separable data distribution. Hence, in this paper, we further extend SDA feature fusion approach in the kernel space and present two solutions to solve the small sample size problem, which are KPCA-SDA and KSDA-GSVD. In KPCA-SDA, we first use KPCA to transform each single modal input space *R^n^* into an *m*-dimensional space, where *m* = rank(*K*), *K* is the centralized Gram matrix. Then SDA is used to fuse the two transformed features and extract discriminative features. In KSDA-GSVD, we directly perform Kernel SDA to fuse multimodal data by applying GSVD to avoid the singular problem.

We evaluate the proposed approaches on two face databases (AR and FRGC), and the PolyU palmprint database, and compare the results with related methods that also tend to extract descriptive features from multimodal data. Experimental results show that our approaches achieve higher recognition rates than compared methods, and also get better verification performance than compared methods. It is worthwhile to point out that, although the proposed approaches are validated on data of two modalities, it could be easily extended to multimodal biometric data recognition.

The rest of this paper is organized as follows: Section 2 describes the related work. Section 3 presents our approach. In Section 4, we present the kernelization of our approach. Experiments and results are given in Section 5 and conclusions are drawn in Section 6.

## 2. Related Work

In this section, we first briefly introduce some typical multimodal biometrics fusion techniques such as pixel level fusion [1,2], Yang's serial and parallel feature level fusion methods [3]. Further, three related methods, which are SDA, KSDA and KPCA, are also briefly reviewed.

### 2.1. Multimodal Fusion Scheme at the Pixel Level

The general idea of pixel level fusion [1,2] is to fuse the input data from multi-modalities in as early as the pixel level, which may lead to less information loss. The pixel level fusion scheme fuses the original input face data vector and palmprint data vector of one person, and then the discriminant features are extracted from the fused dataset. For simplicity and fair comparison, we testified the effectiveness of such scheme by extracting LDA features from the fused set in this paper.

### 2.2. Serial Fusion Strategy and Parallel Fusion Strategy

In [3], Yang *et al.* the authors discussed two strategies to fuse features of two data modes. One is called serial strategy and the other is called parallel strategy. Let *x_i_, y_i_* denote the face feature vector and palmprint feature vector of the *i^th^* person, respectively. The serial fusion strategy obtains the fused features by stacking two vectors into one higher dimensional vector *α_i_, i.e.*:

(1) $$\alpha_{i}=\left[\begin{array}{c} x_{i}\\ y_{i} \end{array}\right]$$

On the other hand, the parallel fusion strategy combines the features into a complex vector *β_i_, i.e.*,

(2) $$\beta_{i}=x_{i}+i\cdot y_{i}$$

Yang *et al.* also pointed out that the fused feature set {*α_i_*} and {*β_i_*} can either be used directly for classification, which is called feature combination, or can be input into a feature extractor to further extract more descriptive features with less redundant information, which is called feature fusion.

### 2.3. Subclass Discriminant Analysis (SDA) and Its Kernelization

Subclass discriminant analysis (SDA) [30] is an extension of LDA, which aims at processing data of one class that form mixture Gaussian distribution. It divides each class into a number of subclasses, and calculates a transform space where the distances between both class means and subclass means are maximized, and distances between samples of each subclass is minimized. SDA redefines the between-class scatter Σ*_B_*, within-class scatter Σ*_W_* as:

(3) $$\sum_{B}=\sum_{i=1}^{C-1}\sum_{j=1}^{H_{i}}\sum_{k=i}^{C}\sum_{l=1}^{H_{k}}p_{\text{ij}}p_{\text{kl}}(\mu_{\text{ij}}-\mu_{\text{kl}}){\left(\mu_{\text{ij}}-\mu_{\text{kl}}\right)}^{T}$$

(4) $$\sum_{W}=\frac{1}{m}\sum_{i=1}^{c}\sum_{j=1}^{H}\sum_{k=i}^{n_{\text{ij}}}(x_{\text{ij}}^{k}-\mu_{\text{ij}}){\left(x_{\text{ij}}^{k}-\mu_{\text{ij}}\right)}^{T}$$

where *H_i_* is the number of subclasses of class *i, p_ij_= n_ij_/n* is the prior of the *j^th^* subclass of class *i, μ_ij_* is the mean of the *j^th^* subclass of class *i*. The advantage of this new definition of between class scatter is that it emphasizes the role of class separability over that of intra-subclass scatter. The optimal solution of SDA is the eigenvectors of matrix (Σ*_W_*)^−1^Σ*_B_* associated with the largest eigenvalues.

Kernel subclass discriminant analysis (KSDA) is the nonlinear extension of SDA based on kernel functions [26]. The main idea of the kernel method is that without knowing the nonlinear feature mapping explicitly, we can work on the feature space through kernel functions. It first maps the input data *x* into a feature space *F* by using a nonlinear mapping Ø. KSDA adopts nonlinear clustering technique to find the underlying distributions of datasets in the kernel space. The between-class scatter matrix $S_{\text{KSDA}}^{\left(b\right)}$ and within-class scatter matrix $S_{\text{KSDA}}^{\left(b\right)}$ of KSDA are defined as:

(5) $$S_{\text{KSDA}}^{\left(b\right)}=\sum_{i=1}^{C-1}\sum_{j=1}^{H_{i}}\sum_{k=i}^{C}\sum_{l=1}^{H_{k}}p_{\text{ij}}p_{\text{kl}}(\bar{\phi_{\text{ij}}}-\bar{\phi_{\text{kl}}}){\left(\bar{\phi_{\text{ij}}}-\bar{\phi_{\text{kl}}}\right)}^{T}$$

(6) $$S_{\text{KSDA}}^{\left(w\right)}=\frac{1}{m}\sum_{i=1}^{c}\sum_{j=1}^{H}\sum_{k=1}^{n_{\text{ij}}}(\phi_{\text{ij}}^{k}-\bar{\phi_{\text{kl}}}){\left(\phi_{\text{ij}}^{k}-\bar{\phi_{\text{kl}}}\right)}^{T}$$

where *ø̄_ij_* indicates the mean vector of *j^th^* subclass of *i^th^* class, *ø̄*is the global mean. Like SDA, KSDA tries to maximize the ratio $\left|V^{T}S_{\text{KSDA}}^{\left(b\right)}V\right|/\left|V^{T}S_{\text{KSDA}}^{\left(m\right)}V\right|$ to find a transformation matrix *V*. The columns of *V* are the eigenvectors corresponding to the largest eigenvalues of ${\left(S_{\text{KSDA}}^{\left(w\right)}\right)}^{-1}S_{\text{KSDA}}^{\left(b\right)}\cdot$

### 2.4. Kernel Principle Component Analysis

In kernel PCA [33], the input data *x* is mapped into a feature space *F* via a nonlinear mapping Ø and then perform a linear PCA in *F*. To be specific, we centralize the mapped data as $\sum_{i=1}^{M}\emptyset (x_{i})=0$ firstly, where *M* is the number of input data. Then the covariance matrix of the mapped data Ø(*x_i_*) is defined as follows:

(7) $$C=1/M\sum_{i=1}^{M}\phi (x_{i})\cdot \phi {\left(x_{i}\right)}^{T}$$

Like PCA, the eigenvalue equation *λV* = *CV* must be solved for eigenvalue *λ* ≥ 0 and eigenvector *V* ∈ *F*\{0}. We can prove that all the solutions *V* lie in the space spanned by Ø(*x*_1_),… Ø (*x_M_*). Therefore, we may consider the equivalent system:

(8) $$\lambda (\phi (x_{k})\cdot V)=(\phi (x_{k}),CV)\text{for all}\;k=1,\ldots M$$

and *V* can be represented as the linear combination of the mapped data Ø(*x_i_*): coefficients *α*_1_,…*α_M_* such that:

(9) $$V=\sum_{i=1}^{M}\alpha_{i}\phi (x_{i})$$

where *α*_1_,…*α_M_* denotes the coefficients. Substituting Equations (8) and (9) into Equations (7), and defining an *M* × *M* matrix *K* by:

(10) $$K_{\text{ij}}=(\phi (x_{i})\phi (x_{j}))$$

we arrive at:

(11) $$\ell \lambda K\alpha =K^{2}\alpha$$

where *α* denotes the column vector with entries *α*_1_,…*α_M_, K* is defined as the kernel matrix. To find solutions of Equation (11) we can solve the equivalent eigenvalue problem as follows:

(12) ℓλα=Kα

for nonzero eigenvalues and obtain the optimal *α*. Finally, we can project mapped Ø (*x_i_*) onto *V* by using *α* to get the KPCA-transformed features [33].

## 3. Subclass Discriminant Analysis (SDA) Based Multimodal Biometric Feature Extraction

In this section, we propose a novel multimodal biometric feature extraction scheme based on SDA. Two solutions are separately introduced to avoid the singular problem in SDA, which are PCA and GSVD. Then we present the algorithm procedures of the proposed SDA-PCA and SDA-GSVD approaches.

### 3.1. Problem Formulation

**Figure 1.**
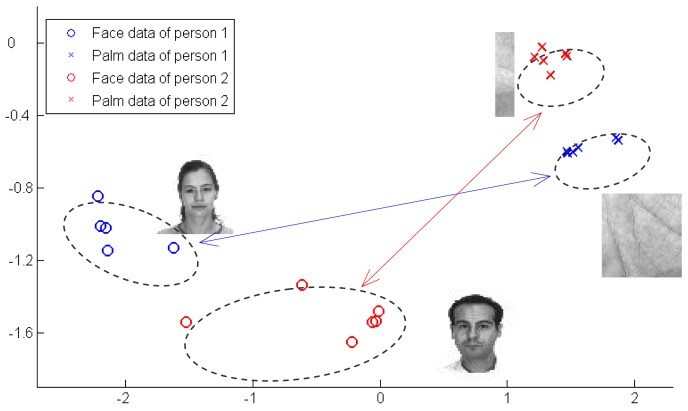
Illustration of mix-Gaussian distribution of face data and the corresponding palmprint data. In this example, data of two persons are presented. Each contains 12 data, including six faces and s palmprints. We perform PCA on original data for demonstration, and the order of data magnitude is 1e4.

For simplicity, we take two typical types of biometric data as examples in this paper. One is the face data, and the other is the palmprint data. From the viewpoint of discrimination, it is quite natural to assume that the overall biometric data one person may be regarded as one class. Moreover, his palmprint and face data can be regarded as two subclasses of this class in the same feature space. An example of two person's face and palmprint samples is shown in Figure 1.

As can be seen from Figure 1, identifier samples of one person show typical mix-Gaussian distribution, *i.e.*, the face data cluster together and form a Gaussian, while the palmprint data form another Gaussian. If we apply traditional LDA, which enforces both of face and palmprint data of one person to cluster together, then data of two persons would be very likely overlap in the embedded space. It is apparent that, in Figure 1, SDA is a better descriptor of such a data distribution.

Let $x_{i1}^{k}$ and $x_{i2}^{k}$ be the *k^th^* face sample and palmprint sample of person *i*, respectively; *n_c_* represent the sample number of each subclass. Then we construct the between-subclass scatter matrix *S_B_* and within-subclass scatter matrix *S_W_* as follows:

(13a) $$S_{B}=\sum_{i=1}^{c-1}\sum_{j=1}^{2}\sum_{k=i+1}^{c}\sum_{l=1}^{2}p_{\text{ij}}p_{\text{kl}}(\mu_{\text{ij}}-\mu_{\text{kl}}){\left(\mu_{\text{ij}}-\mu_{\text{kl}}\right)}^{T}$$

(13b) $$S_{W}=\frac{1}{N}\sum_{i=1}^{c}\sum_{j=1}^{2}\sum_{k=1}^{nc}(x_{\text{ij}}^{k}-\mu_{\text{ij}}){\left(x_{\text{ij}}^{k}-\mu_{\text{ij}}\right)}^{T}$$

where *N* = *c* × *n*_c_, *p_ij_* = *p_kl_* = *n_c_/N*, $\mu_{\text{ij}}=\sum_{k=1}^{nc}x_{\text{ij}}^{k}/n_{c}\cdot$

Let be the optimal transform vector to be calculated, and then it can be obtained by:

(14) $$\underset{w}{\max}\frac{w^{T}S_{B}w}{w^{T}S_{B}w}$$

The within-class matrix *S_W_* is usually singular, and the solution cannot be calculated directly. We present two solutions below to solve this problem, *i.e.*, SDA-PCA and SDA-GSVD.

### 3.2. SDA-PCA

The first solution is to first apply PCA to project each image $x_{i1}^{k}$ into a lower dimensional space, and then apply SDA to do feature extraction. By employing the Lagrange multipliers method to solve the optimization problem (15), we could obtain the optimal solution *W_SDA_, i.e.*, the eigenvectors of matrix (*S_W_*)^−1^*S_B_* associated with the largest eigenvalues.

Based on Formula (14), the rank of *S_W_* is *n – 2c*, where *n* represents the total number of training samples (including face and palmprint images), and *c* represents the number of persons. Therefore, we can project original samples into a subspace whose dimension is no more than *n – 2c*, and then apply SDA to extract features.

Let $W_{\text{PCA}}^{1},W_{\text{PCA}}^{2}$ separately denote the initial PCA transformations of the sample set of each modal, and *W_SDA_* denote the later SDA transform. Then the final transformations for each modal are expressed as:

(15) $${\hat{W}}_{1}=W_{\text{PCA}}^{1}W_{\text{SDA}}$$

(16) $${\hat{W}}_{2}=W_{\text{PCA}}^{2}W_{\text{SDA}}$$

After the optimal transformations *Wˆ*_1_ and *Wˆ*_2_ are obtained, we project the face sample $x_{i1}^{k}$ and palmprint sample $x_{i2}^{k}$ on them:

(17) $$y_{i1}^{k}={\hat{W}}_{1}^{T}x_{i1}^{k},y_{i2}^{k}={\hat{W}}_{2}^{T}x_{i2}^{k}$$

Then, features derived from face and palmprint are fused used using serial fusion strategy and used for classification:

(18) $$y_{i}^{k}=\left[\begin{array}{c} y_{i1}^{k}\\ y_{i2}^{k} \end{array}\right]$$

### 3.3. SDA-GSVD

While PCA is a popular way to overcome the singular problem and accelerate computation, it may cause information loss. Therefore, we present a second way to overcome the singularity problem by employing GSVD. First, we rewrite the between-class scatter matrix and within-class scatter matrix as follows:

(19) $$S_{B}=H_{b}H_{b}^{T},S_{w}=H_{w}H_{w}^{T}$$

*H_b_* is obtained by transforming formula (13) as follows:

(20) $$\begin{array}{ll} S_{B} & =\sum_{i=1}^{c-1}\sum_{j=1}^{2}\sum_{k=i+1}^{c}\sum_{l=1}^{2}p_{\text{ij}}p_{\text{kl}}(\mu_{\text{ij}}-\mu_{\text{kl}}){\left(\mu_{\text{ij}}-\mu_{\text{kl}}\right)}^{T}\\ & =p_{\text{ij}}p_{\text{kl}}\sum_{i=1}^{c-1}\sum_{j=1}^{2}\left[2(c-i)\mu_{\text{ij}}-\sum_{k=i+1}^{c}\sum_{l=1}^{2}\mu_{\text{kl}}\right]\cdot {\left[2(c-i)\mu_{\text{ij}}-\sum_{k=i+1}^{c}\sum_{l=1}^{2}\mu_{\text{kl}}\right]}^{T} \end{array}$$

Compared with Equation (21) *H_b_* is defined as:

(21) $$H_{b}=[H_{(c-1)1},H_{(c-1)2},H_{(c-2)1},\ldots ,H_{11},H_{12}]$$

where. $H_{(c-m)n}=2(c-N)\mu_{mn}-\sum_{k=m+1}^{c}\sum_{l=1}^{2}\mu_{\text{kl}}$

According to Equation (14), we can easily achieve *H_w_*:

(22) $$H_{w}={\left[x_{\text{ij}}^{1}-\mu_{\text{ij}},x_{\text{ij}}^{2}-\mu_{\text{ij}},\ldots x_{\text{ij}}^{n_{c}}-\mu_{\text{ij}}\right]}_{i=1,\ldots c,j=1,2}$$

Then, we employ GSVD [31,32] to calculate the optimal transform, and the procedures are given in Algorithm 1.

**Algorithm 1.** Procedures of GSVD based LDA.**Step 1:** Define matrix *K =* [*H_b_, H_w_*]*^T^*, and compute the complete orthogonal decomposition 
$P^{T}KQ=\left\{\begin{array}{cc} R & 0\\ 0 & 0 \end{array}\right\}$**Step 2:** Compute *G* by performing SVD on matrix P(1:*c*,1:*t*), *i.e.,U^T^P*(1:*c*,1:*t*)G=Σ*_A_*, where *t* is the rank of *K*.**Step 3:** Compute matrix . 
$M=Q\left\{\begin{array}{cc} R^{-1}G & 0\\ 0 & I \end{array}\right\}$. Put the first *c −* 1 columns of *M* into matrix *W*. Then, *W* is the optimal transform matrix.

Then, face data $x_{i1}^{k}$ and palmprint data $x_{i2}^{k}$ are separately projected on *W* and fused using serial fusion strategy:

(23) $$y_{i}^{k}=\left[\begin{array}{c} y_{i1}^{k}\\ y_{i2}^{k} \end{array}\right]=\left[\begin{array}{c} {\hat{W}}^{T}x_{i1}^{k}\\ {\hat{W}}^{T}x_{i2}^{k} \end{array}\right]$$

$y_{i}^{k}$ is then used for classification.

### 3.4. Algorithmic Procedures

In this section, we summarize the complete algorithmic procedures of the proposed approach. In practice, if the dimension of two biometric data $x_{i1}^{k}$ and $x_{i2}^{k}$ are not equal, we could simply pad the lower-dimensional vector with zeros until its dimension is equal to the other one before fusing them using SDA. In case of SDA-PCA, after PCA projection, it is easy guarantee that $x_{i1}^{k}$ and $x_{i2}^{k}$ have the same dimension if we select the same number of principal components for them.

**Figure 2.**
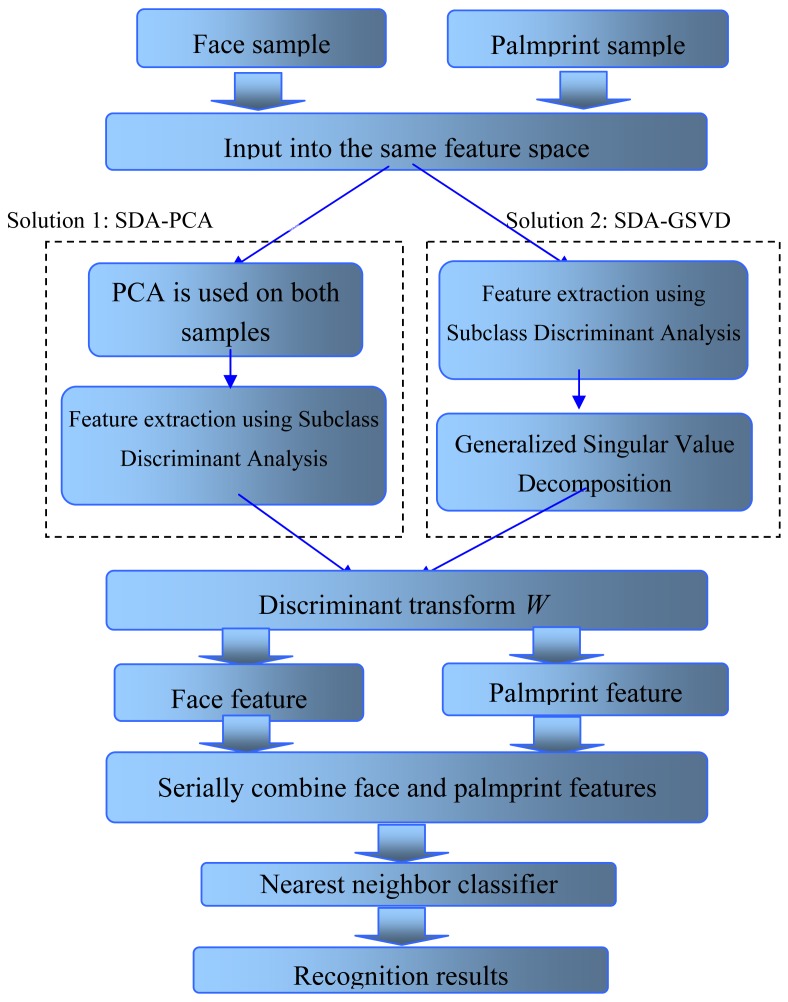
The complete procedures of SDA based multimodal feature extraction.

Figure 2 displays the complete procedure of the proposed approach for multimodal biometric recognition. It is worthwhile to note that, on one hand, our approach outputs features of each modal separately, which is convenient for later processing; on the other hand, discriminative information of different modals have been initially fused in the extraction process, since their features are extracted from the same input space and the transformed space also consider the distribution of data of other modals. Therefore, we think this approach can effectively obtain fused discriminative information from multimodal data.

## 4. SDA Kernelization Based Multimodal Biometric Feature Extraction

In this section, we provide the nonlinear extensions of two SDA based multimodal feature extraction approaches, which are named KPCA-SDA and KSDA-GSVD. In KPCA-SDA, we first apply Kernel PCA on each single modal before performing SDA. While in KSDA-GSVD, we directly perform Kernel SDA to fuse multimodal data by applying GSVD to avoid the singular problem.

### 4.1. KPCA-SDA

In this subsection, the SDA-PCA approach is performed in a high dimension space by using the kernel trick. We realized the KPCA-SDA in the following steps:

1. Nonlinear mapping.
  Let ∅: *R^d^* → *F* denote a nonlinear mapping. The original samples $x_{i1}^{k}$ and $x_{i2}^{k}$ of two modalities (face and palmprint) are injected into *F* by $\emptyset :x_{i1}^{k}\to \emptyset (x_{i1}^{k}),x_{i2}^{k}\to \emptyset (x_{i2}^{k})$. We obtain two sets of mapped samples $\Psi_{1}=\{\emptyset (x_{11}^{1}),\emptyset (x_{11}^{2}),\ldots ,\emptyset (x_{c_{1}}^{n_{c}})\}$, $\Psi_{2}=\{\emptyset (x_{12}^{1}),\emptyset (x_{12}^{2}),\ldots ,\emptyset (x_{c_{2}}^{n_{c}})\}$
2. Perform KPCA for each single modal database.
  For the *j^th^* modal, we perform KPCA by maximizing the following equation:
  (24) $$J(w_{\text{kpca}}^{j^{\phi }})={w_{\text{kpca}}^{j^{\phi }}}^{T}S_{t}^{j\phi }S_{\text{kpca}}^{j\phi }$$
  where $S_{t}^{j\phi }=\sum_{i=1}^{c}\sum_{k=1}^{n_{c}}\left(\phi (x_{\text{ij}}^{k})-m_{j}^{\phi }\right){\left(\phi (x_{\text{ij}})-m_{j}^{\phi }\right)}^{T}$, and $m_{j}^{\phi }$ is the global mean of the *j^th^* modal database in the kernel space.
  According to the kernel reproducing theory [34], the projection transformation $w_{\text{kpca}}^{j\emptyset }$ in *F* can be linearly expressed by using all the mapped samples:
  (25) $$w_{\text{kpca}}^{j^{\phi }}=\sum_{i=1}^{c}\sum_{k=1}^{n_{c}}\alpha_{\text{ij}}^{k}\phi (x_{\text{ij}}^{k})=\Psi_{j}\alpha_{j}$$
  where $\alpha_{j}={\left(\alpha_{1j}^{1},\alpha_{1j}^{2},\cdots ,\alpha_{cj}^{nc}\right)}^{T}$ is a coefficient matrix.
  Substituting Equation (26) into Equation (25), we have:
  (26) $$J(w^{j^{\phi }})=\alpha_{j}^{T}\Psi_{j}^{T}\Psi_{j}\Psi_{j}^{T}\Psi_{j}\alpha_{j}=\alpha_{j}^{T}K^{j}K^{jT}\alpha_{j}$$
  where $K^{j}=\Psi_{j}^{T}\Psi_{j}$, which indicates an *N* × *N* non-symmetric kernel matrix whose element is $K_{m,n}^{j}=\langle \phi (x_{j}m),\phi (x_{j}n)\rangle$, where *N* denotes the total number of the samples, *x_j_m* denotes the *m^th^* sample of the *j^th^* modal database.
  The solution of Equation (27) is equivalent to the eigenvalue problem:
  (27) $$\lambda^{j}\alpha_{j}=K_{j}K_{j}^{T}\alpha_{j}$$
  The optimal solutions *α_j_* = (*α_j_*_1_, *α_j_*_2_,…, *α_j_*_(_*_N-c_*_)_)*^T^* are the eigenvectors corresponding to *N − c* largest eigenvalues of $K_{j}K_{j}^{T}$. We project the mapped training sample set Ψ*_j_* on $w_{\text{kpca}}^{j^{\emptyset }}$ by:
  (28) $$Z_{\text{KPCA}}^{j^{\phi }}={w_{\text{kpca}}^{j^{\phi }}}^{T}\Psi_{j}=\alpha_{j}^{T}\Psi_{j}^{T}\Psi_{j}=\alpha_{j}^{T}K_{j}$$
3. Calculate kernel discriminant vectors in the KPCA transformed space.
  By using the KPCA transformed sample set $Z_{K\text{PCA}}^{j^{\emptyset }}$, we reformulate Equations (13) and (14) as:
  (29) $$S_{B}^{\phi }=\sum_{i=1}^{c-1}\sum_{j=1}^{2}\sum_{k=i+1}^{c}\sum_{l=1}^{2}p_{\text{ij}}p_{\text{kl}}(\mu_{\text{ij}}^{\phi }-\mu_{\text{kl}}^{\phi }){\left(\mu_{\text{ij}}^{\phi }-\mu_{\text{kl}}^{\phi }\right)}^{T}$$
  (30) $$S_{W}^{\phi }=\frac{1}{N}\sum_{i=1}^{c}\sum_{j=1}^{2}\sum_{k=1}^{n_{c}}(z_{\text{ij}}^{k^{\phi }}-\mu_{\text{kl}}^{\phi }){\left(z_{\text{ij}}^{k^{\phi }}-\mu_{\text{kl}}^{\phi }\right)}^{T}$$
  where $Z_{\text{ij}}^{k^{\emptyset }}$ is the sample in $Z_{K\text{PCA}}^{j^{\emptyset }}$ and $\mu_{\text{ij}}^{\phi }=\sum_{k=1}^{n_{c}}z_{\text{ij}}^{k^{\phi }}/n_{c}$.
  We can obtain a set of nonlinear discriminant vectors $W_{\text{SDA}}^{\phi }$, *i.e.*, the eigenvector of matrix ${\left(S_{W}^{\emptyset }\right)}^{-1}S_{B}^{\emptyset }$ associated with the largest eigenvalues.
4. Construct the nonlinear projection transformation and do classification.
  We then construct the nonlinear projection transformation *W^jØ^* as:
  (31) $$W^{j^{\phi }}=w_{\text{kpca}}^{j^{\phi }}W_{\text{SDA}}^{\phi }$$
  After the optimal transform *W^jØ^* is obtained, the fused features can be generated as:
  (32) $$y^{\phi }=\left[\begin{array}{cc} W^{1^{\phi T}} & \Psi_{1}\\ W^{2^{\phi T}} & \Psi_{2} \end{array}\right]$$

### 4.2. KSDA-GSVD

In this subsection, the SDA-GSVD is performed in a high dimension space by using the kernel trick. Given two sets of mapped samples $\Psi_{1}=\{\emptyset (x_{11}^{1}),\emptyset (x_{11}^{2}),\ldots ,\emptyset (x_{c_{1}}^{n_{c}})\}$, $\Psi_{2}=\{\emptyset (x_{12}^{1}),\emptyset (x_{12}^{2}),\ldots ,\emptyset (x_{c_{2}}^{n_{c}})\}$, that correspond to face and palmprint modalities, respectively. Afterwards, *H_b_* and *H_w_* are recalculated in the kernel space:

(33) $$H_{b}^{\phi }=[H_{(c-1)1}^{\phi },H_{(c-1)2}^{\phi },H_{(c-2)1}^{\phi },\cdots ,H_{11}^{\phi },H_{12}^{\phi }]$$

(34) $$H_{w}^{\phi }={\left[\phi (x_{\text{ij}}^{1})-\mu_{\text{ij}}^{\phi },\phi (x_{\text{ij}}^{2})-\mu_{\text{ij}}^{\phi },\ldots \phi (x_{\text{ij}}^{n_{c}})-\mu_{\text{ij}}^{\phi }\right]}_{i=1,\ldots c,j=1,2}$$

(35) $$\text{where}\;H_{(c-m)n}^{\phi }=2(c-N)\mu_{mn}^{\phi }-\sum_{k=m+1}^{c}\sum_{l=1}^{2}\mu_{\text{kl}}^{\phi },\text{and}\;\mu_{\text{ij}}=\sum_{k=1}^{n_{c}}\phi (x_{\text{ij}}^{k})/n_{c}$$

Then, we apply GSVD to calculate the optimal transformation so that the singular problem is avoided. The procedures are precisely introduced in Algorithm 1. When the optimal *WØ* is obtained, the fused features can be generated as:

(36) $$Y^{\phi }=\left[\begin{array}{c} y_{i1}^{k}\\ y_{i2}^{k} \end{array}\right]=\left[\begin{array}{c} {\hat{W}}^{\phi^{T}}\phi (x_{i1}^{k})\\ {\hat{W}}^{\phi^{T}}\phi (x_{i2}^{k}) \end{array}\right]=\left[\begin{array}{c} {\hat{W}}^{\phi^{T}}\Psi_{1}\\ {\hat{W}}^{\phi^{T}}\Psi_{2} \end{array}\right]$$

Finally, the nearest neighbor classifier with cosine distance is employed to perform classification.

## 5. Experiments

In this section, we compare the proposed multimodal feature extraction approaches with single modal method and several representative multimodal biometric fusion methods. The identification and verification performance of our approaches and other compared methods is evaluated on two face databases and one palmprint database.

### 5.1. Introduction of Databases

**Figure 3.**
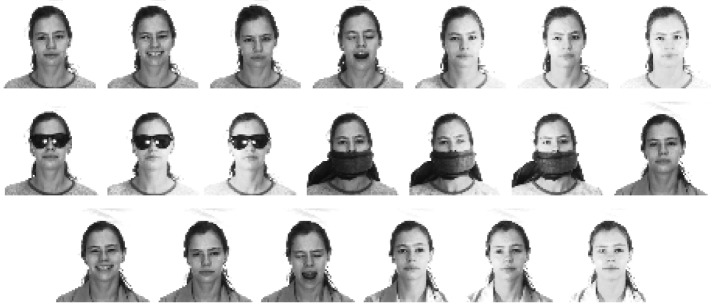
Demo images of one subject from the AR face database.

Two public face databases (AR and FRGC) and one public palmprint database (PolyU palmprint database) are employed to testify our proposed approaches. The AR face database [35] contains over 4,000 color face images of 126 people (70 men and 56 women), including frontal views of faces with different facial expressions, under different lighting conditions and with various occlusions. Most of the pictures were taken in two sessions (separated by two weeks). Each session yielded 13 color images, with 119 individuals (65 men and 54 women) participating in each session. We selected images from 119 individuals for use in our experiment for a total number of 3,094 (=119 × 26) samples. All color images are transformed into gray images and each image was scaled to 60 × 60 with 256 gray levels. Figure 3 illustrates all of the samples of one subject.

**Figure 4.**
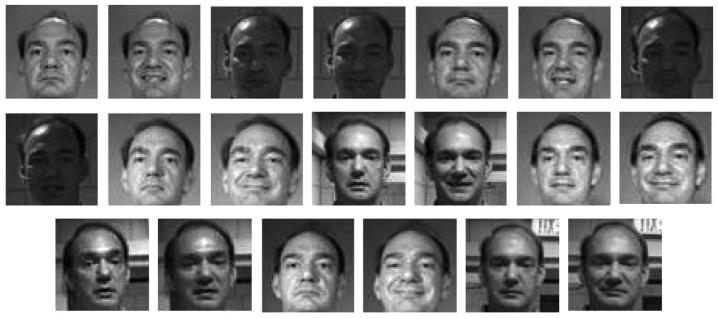
Demo images of one subject from the FRGC face database.

The FRGC database [36] contains 12,776 training images that consist of both controlled images and uncontrolled images, including 222 individuals, each 36–64 images for the FRGC Experiment 4. The controlled images have good image quality, while the uncontrolled images display poor image quality, such as large illumination variations, low resolution of the face region, and possible blurring. It is these uncontrolled factors that pose the grand challenge to face recognition performance. We use the training images of the FRGC Experiment 4 as our database. We choose 36 images of each individual and then crop every image to the size of 60 × 60. All images of one subject are shown in Figure 4.

**Figure 5.**
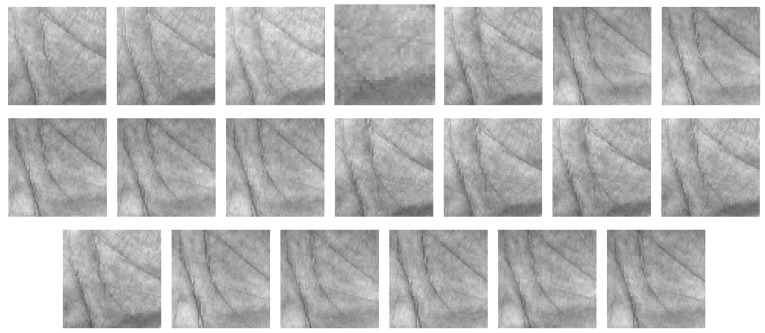
Demo images of one subject from the PolyU palmprint database.

The palmprint database [37,38], which is provided by the Hong Kong Polytechnic University (HK PolyU), collected palmprint images from 189 individuals. Around 20 palmprint images from each individual were collected in two sessions, where around 10 samples were captured in the first session and the second session, respectively. Therefore, the database contains a total of 3,780 images from 189 palms. In order to reduce the computational cost, each subimage was compressed to 60 × 60. We took these subimages as palmprint image samples for our experiments. All cropped images of one subject in Figure 5.

In order to testify the proposed fusion techniques, in the experiment which we fuse AR database and PolyU palmprint database, we choose 119 subjects from both face and palmprint database, and each class contains 20 samples. Similarly, in the experiment which we fuse FRGC database and PolyU palmprint database, we choose 189 subjects from both face and palmprint database, and each class contains 20 samples. We assume that samples of one subject in the palmprint database correspond to the samples of one subject in the face database. For the AR face database and PolyU palmprint database, we randomly select eight samples from each person (four face samples from AR database and four palmprint samples from PloyU database) for training, while use the rest for testing. For the FRGC face database and PolyU palmprint database, we randomly select six samples from each person (three face samples from FRGC database and three palmprint samples from PloyU database) for training, while use the rest for testing. We run all compared methods 20 times. In our experiments, we consider the Gaussian kernel $k(x,y)=\exp(-{\left\|x-y\right\|}^{2}/2\delta_{i}^{2})$ for the compared kernel methods, and set the parameter *δ_i_* = *i* × *δ ,i* ∈ 1,···,20, where *δ* is the standard deviation of training data set. For each compared kernel method, the parameter *i* was selected such that the best classification performance was obtained.

### 5.2. Experimental Identification Results

**Figure 6.**
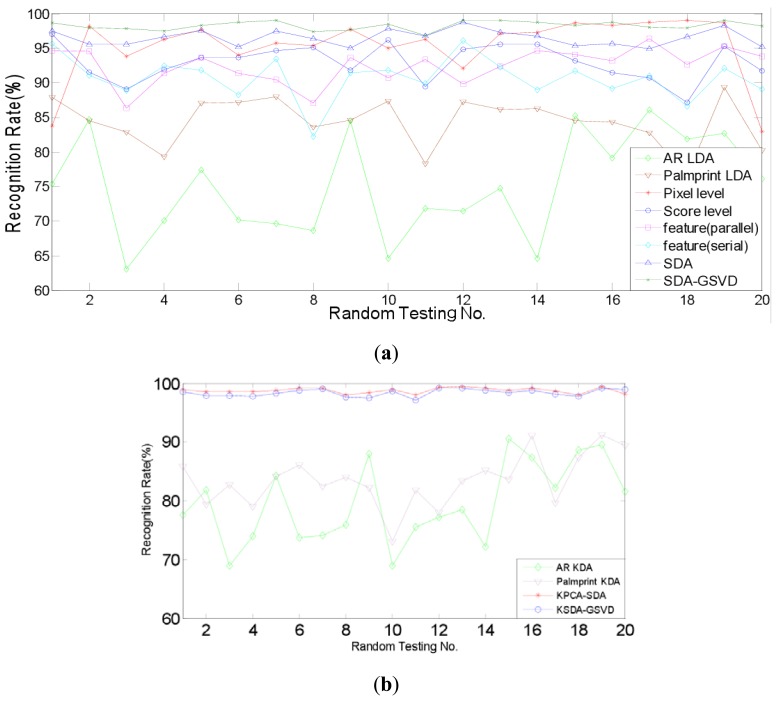
Recognition rates of compared methods on AR face and PolyU palmprint databases: (**a**) Linear methods; (**b**) Nonlinear methods.

**Figure 7.**
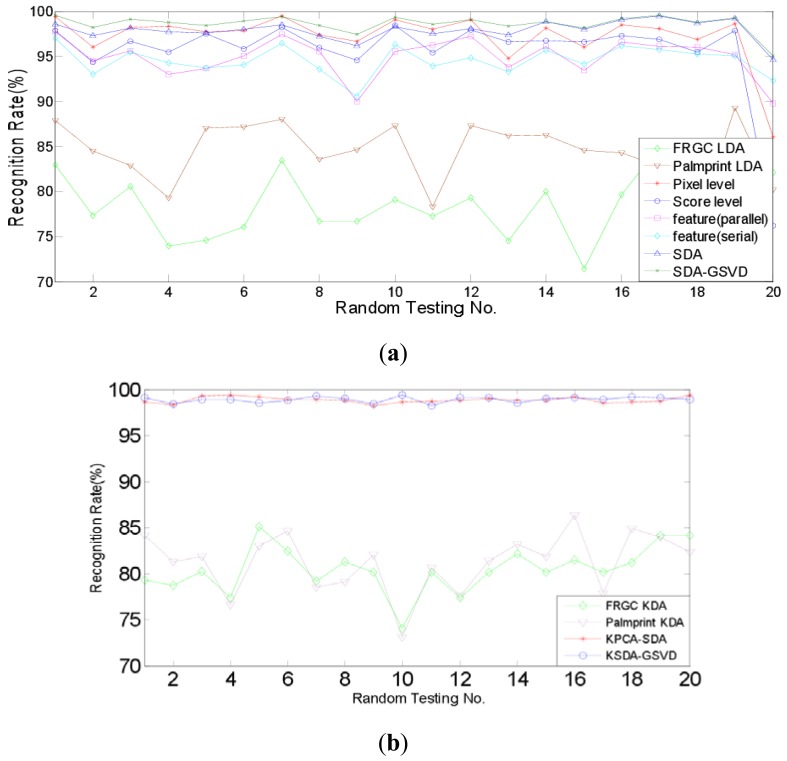
Recognition rates of compared methods on FRGC face and PolyU palmprint databases: (**a**) Linear methods; (**b**) Nonlinear methods.

Firstly, the identification experiments are conducted. Identification is a one-to-many comparison which aims to answer the question of “who is this person?” We compare the identification performance of two proposed approaches, *i.e.*, SDA-PCA (which is abbreviated to SDA here), SDA-GSVD, with single modal recognition method using traditional LDA, a representative pixel level fusion method [1], parallel and serial feature level fusion [3], and score level fusion method using the sum rule [7], respectively. Further, we compare the proposed kernelizaion methods (KPCA-SDA and KSDA-GSVD), with single modal recognition method using KDA. Figures 6 and 7 show the recognition rates of 20 random tests of our approaches and other compared methods: (a) SDA, SDA-GSVD, LDA (single modal), Pixel level fusion, parallel feature fusion, Serial feature fusion and Score level fusion; (b) KPCA-SDA, KSDA-GSVD and KDA (single modal). The average recognition rates are given in Tables 1 and 2, which correspond to the figures above.

Table 1 shows that on the AR and PolyU palmprint databases, SDA and SDA-GSVD perform better than other compared linear methods. It also shows that KPCA-SDA and KSDA-GSVD achieve better recognition results than KDA (single modal). Compared with the single modal LDA, pixel level fusion, parallel feature fusion, parallel feature fusion, serial feature fusion and score level fusion, SDA improves the average recognition rate at least by 3.53% (=98.23%–92.99%), SDA-GSVD improves the average recognition rate at least by 5.24% (=98.23%–92.99%). And the average recognition rate of KPCA-SDA is at least 15.29% (=98.74%–83.45%) higher than that of KDA (single modal), and the average recognition rate of KSDA-GSVD is at least 15.7% (=99.15%–83.45%) higher than that of KDA (single modal). Table 2 shows a similar phenomenon on the FRGC and PolyU palmprint databases. SDA boosts the average recognition rate at least by 0.85% (=98.06%–97.21%), and SDA-GSVD boosts the average recognition rate at least by 1.40% (=98.61%–97.21%) than other linear methods. The average recognition rate of KPCA-SDA is at least 17.59% (=98.82–81.23) higher than that of KDA (single modal), and the average recognition rate of KSDA-GSVD is at least 17.79% (=99.02%–81.23%) higher than that of KDA (single modal).

### 5.3. Experimental Results of Verification

Verification is a one-to-one comparison which aims to answer the question of “whether the person is one he/she claims to be”. In the verification experiments, we show the receiver operating characteristic (ROC) curves, which plot the false rejection rate (FRR) *versus* the false accept rate (FAR), to report the verification performance. There is a tradeoff between the FRR and the FAR. It is possible to reduce one of them with the risk of increasing the other one. Thus the curve which is called receiver operating characteristic (ROC) reflects the tradeoff between the FAR and FRR, and FRR is plotted as a function of FAR.

**Figure 8.**
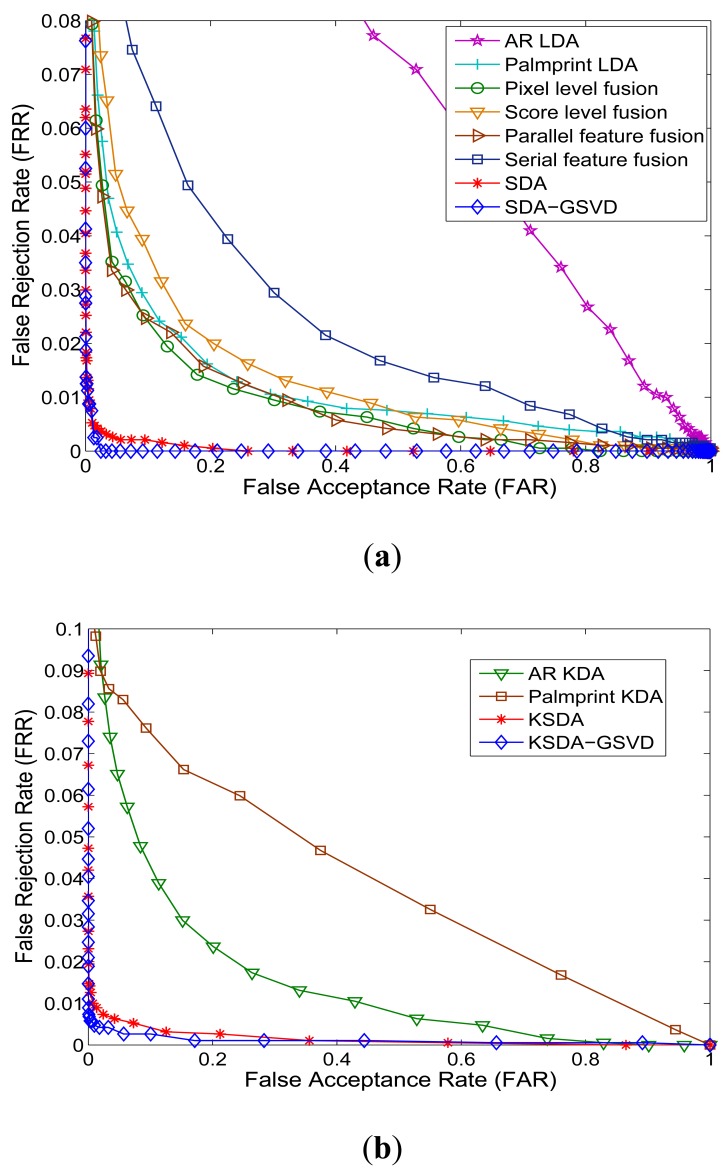
ROC curves of all compared methods on AR face and PolyU palmprint databases: (**a**) Linear methods; (**b**) Nonlinear methods.

**Figure 9.**
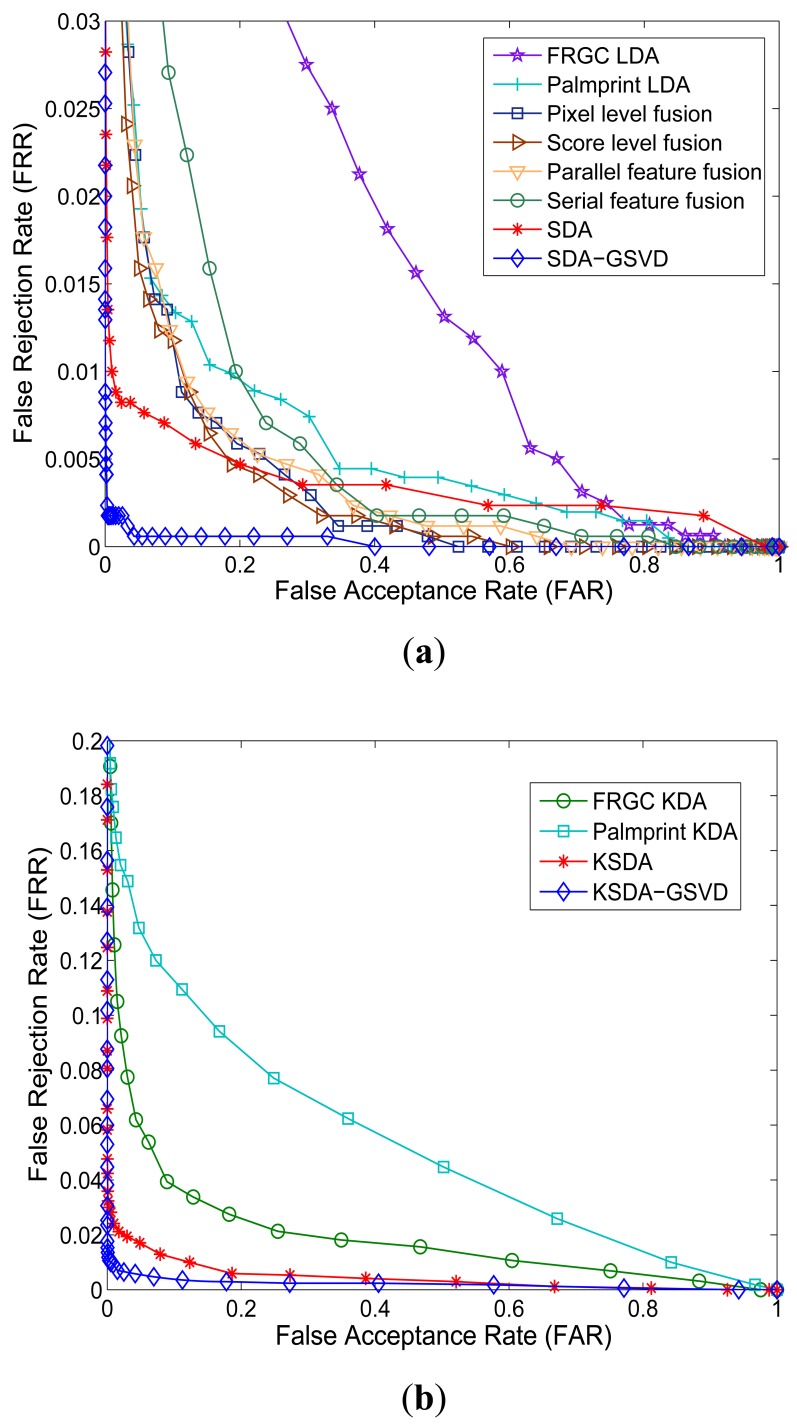
ROC curves of all compared methods on FRGC face and PolyU palmprint databases: (**a**) Linear methods; (**b**) Nonlinear methods.

Figures 8 and 9 show the Receiver Operating Characteristic (ROC) curves of our approaches and other compared methods on different databases. Table 3 shows the equal error rate (EER) of all compared methods. From the ROC curves shown in Figures 8–9 and the results listed in Table 3, we can see that our SDA based feature extraction approaches attains a significantly low EER (a point on the ROC curve where FAR is equal to FRR) than other representative multimodal fusion methods, including pixel level fusion method, score level fusion method and feature level fusion methods. On the AR face and PolyU palmprint databases, the lowest EER of related methods is 3.71%, while the EER of our approaches are all below 1%. And our KSDA-GSVD approach obtains the lowest EER 0.56% among all compared methods. On the FRGC face and PolyU palmprint databases, the lowest EER of other methods is 2.62%, while the EER of ours are all below 2%. Especially, the proposed SDA-GSVD approach gets the lowest EER that is 0.28%. The above experimental results demonstrate the superiority of our approaches.

## 6. Conclusions

In this paper, we present novel multimodal biometric feature extraction approaches using subclass discriminant analysis (SDA). Considering the nonsingularity requirements, we present two ways to overcome this problem. The first is to initially do principle component analysis before SDA, and the second is to employ generalized singular value decomposition (GSVD) to directly obtain the solution. Further, we present the kernel extensions (KPCA-SDA and KSDA-GSVD) for multimodal biometric feature extraction. We perform the experiments on two public face databases (*i.e.*, AR face database and FRGC database) and the PolyU palmprint database. In designing the experiments, we firstly do extraction on the AR and palmprint database, secondly on the FRGC and palmprint database. Compared with several representative linear and nonlinear multimodal biometrics recognition methods, the proposed approaches acquire better identification and verification performance. In particular, the proposed KSDA-GSVD approach performs best on all the databases.

## Figures and Tables

**Table 1.** Average recognition rates of compared methods on AR face and PolyU palmprint databases

**AR and palmprint**		**Average recognition rates (%)**
Single modal recognition	AR LDA	75.09 ± 7.39
	Palmprint LDA	82.26 ± 3.50
Multimodal recognition	Pixel level fusion [1]	95.35 ± 4.50
	Parallel feature fusion [3]	92.48 ± 2.61
	Serial feature fusion [3]	90.71 ± 3.06
	Score level fusion [7]	92.99 ± 2.63
	SDA based feature extraction	96.52 ± 1.16
	SDA-GSVD based feature extraction	**98.23** ± **0.68**

(**a**) Linear methods		

**AR and palmprint**		**Average recognition rates (%)**

Single modal recognition	AR KDA	79.50 ± 6.83
	Palmprint KDA	83.45 ± 4.47
Multimodal recognition	KPCA-SDA	98.74 ± 0.45
	KSDA-GSVD	**99.15** ± **0.63**

(**b**) Nonlinear methods		

**Table 2.** Average recognition rates of compared methods on FRGC face and PolyU palmprint databases.

**FRGC and palmprint**		**Average recognition rates (%)**
Single modal recognition	FRGC LDA	78.26 ± 4.53
	Palmprint LDA	80.22 ± 3.26
Multimodal recognition	Pixel level fusion [1]	97.21 ± 2.89
	Parallel feature fusion [3]	94.92 ± 2.17
	Serial feature fusion [3]	94.54 ± 1.57
	Score level fusion [7]	95.59 ± 4.70
	SDA based feature extraction	98.06 ± 1.09
	SDA-GSVD based feature extraction	**98.61** ± **0.99**

(**a**) Linear methods		

**FRGC and palmprint**		**Average recognition rates (%)**

Single modal recognition	AR KDA	80.44 ± 2.57
	Palmprint KDA	81.23 ± 3.26
Multimodal recognition	KPCA-SDA	98.82 ± 0.32
	KSDA-GSVD	**99.02** ± **0.31**

(**b**) Nonlinear methods		

**Table 3.** The equal error rate (EER) of all compared methods on different databases.

**Method**		**AR and PalmprintEER (%)**	**FRGC and Palmprint EER (%)**
Single modal recognition	Face LDA	15.45	8.13
	Palmprint LDA	4.32	3.14
	Face KDA	6.13	5.72
	Palmprint KDA	8.36	10.85
Multimodal recognition	Pixel level fusion [1]	3.95	3.25
	Parallel feature fusion [3]	3.71	3.27
	Serial feature fusion [3]	7.84	4.41
	Score level fusion [7]	5.12	2.62
	SDA based feature extraction	0.83	1.05
	SDA-GSVD based feature extraction	0.72	**0.28**
	KSDA based feature extraction	0.87	1.90
	KSDA-GSVD based feature extraction	**0.56**	0.84
